# Leveraging the transcriptome to further our understanding of GWAS findings: eQTLs associated with genes related to LDL and LDL subclasses, in a cohort of African Americans

**DOI:** 10.3389/fgene.2024.1345541

**Published:** 2024-02-07

**Authors:** Malak Abbas, Ana Diallo, Gabriel Goodney, Amadou Gaye

**Affiliations:** ^1^ National Human Genome Research Institute, National Institutes of Health, Bethesda, MD, United States; ^2^ School of Nursing, Virginia Commonwealth University, Richmond, VA, United States

**Keywords:** LDL, eQTL, transcriptome, African American (AA), GWAS

## Abstract

**Background:** GWAS discoveries often pose a significant challenge in terms of understanding their underlying mechanisms. Further research, such as an integration with expression quantitative trait locus (eQTL) analyses, are required to decipher the mechanisms connecting GWAS variants to phenotypes. An eQTL analysis was conducted on genes associated with low-density lipoprotein (LDL) cholesterol and its subclasses, with the aim of pinpointing genetic variants previously implicated in GWAS studies focused on lipid-related traits. Notably, the study cohort consisted of African Americans, a population characterized by a heightened prevalence of hypercholesterolemia.

**Methods:** A comprehensive differential expression (DE) analysis was undertaken, with a dataset of 17,948 protein-coding mRNA transcripts extracted from the whole-blood transcriptomes of 416 samples to identify mRNA transcripts associated with LDL, with further granularity delineated between small LDL and large LDL subclasses. Subsequently, eQTL analysis was conducted with a subset of 242 samples for which whole-genome sequencing data were available to identify single-nucleotide polymorphisms (SNPs) associated with the LDL-related mRNA transcripts. Lastly, plausible functional connections were established between the identified eQTLs and genetic variants reported in the GWAS catalogue.

**Results:** DE analysis revealed 1,048, 284, and 94 mRNA transcripts that exhibited differential expression in response to LDL, small LDL, and large LDL, respectively. The eQTL analysis identified a total of 9,950 significant SNP-mRNA associations involving 6,955 SNPs including a subset 101 SNPs previously documented in GWAS of LDL and LDL-related traits.

**Conclusion:** Through comprehensive differential expression analysis, we identified numerous mRNA transcripts responsive to LDL, small LDL, and large LDL. Subsequent eQTL analysis revealed a rich landscape of eQTL-mRNA associations, including a subset of eQTL reported in GWAS studies of LDL and related traits. The study serves as a testament to the important role of integrative genomics in unraveling the enigmatic GWAS relationships between genetic variants and the complex fabric of human traits and diseases.

## Introduction

The mechanisms underlying Genome-Wide Association Study (GWAS) variants remain incompletely understood. While GWAS successfully identifies genetic loci associated with various traits and diseases, the precise molecular pathways through which these variants exert their effects often remain elusive.

Expression quantitative trait loci (eQTL) analysis can play a crucial role in enhancing our understanding of the genetic basis of complex traits and diseases identified through GWAS. While GWAS pinpoints genetic variants associated with specific phenotypes, eQTL analysis enables the exploration of how these variants influence gene expression. By identifying regulatory variants in coding and non-coding regions of the genome, eQTL analysis sheds light on the impact variants have on the expression of nearby or distant genes and their functional consequences in relation to GWAS findings. This integration of eQTL data with GWAS outcomes allows for the elucidation of underlying biological pathways, aiding in the prioritization of candidate genes, identification of potential therapeutic targets, and refinement of disease mechanisms. Notable consortia like the GTEx Consortium and eQTLGen Consortium (Vosa et al., 2021) have demonstrated the utility of eQTL analysis in bridging the gap between genetic associations and functional insights.

Lipoprotein quantification, traditionally focused on cholesterol levels, is undergoing reassessment due to emerging evidence emphasizing the significance of particle number and size. Recent studies suggest that particle concentration, as opposed to cholesterol content, may offer a more accurate reflection of the atherogenic potential of lipoproteins. Notably, small dense low-density lipoprotein (LDL) particles have been shown to have a higher association with atherosclerosis risk than their larger, more buoyant counterparts (Otvos et al., 2002; Cromwell et al., 2007). This nuanced perspective on lipoprotein particles enables a more precise risk assessment for cardiovascular diseases, with substantial implications for personalized approaches to lipid management in medicine.

This project leverages the whole-blood transcriptome to conduct an unbiased eQTL scan of the genome and identify Single-Nucleotide Polymorphisms (SNP) associated with messenger RNAs (mRNA) involved in pathways relevant for LDL cholesterol metabolism and hence provide plausible mechanistic links between GWAS-reported SNPs and LDL. The analysis also considered small and large LDL because those lipoprotein particles provide a more nuanced understanding of cardiovascular risk, metabolic disorders, and lipid metabolism; knowledge that leads to improved personalized treatment strategies, and advancements in the prevention and management of cardiovascular diseases.

Our study, centered on an African American cohort, adds to a broader body of research encompassing diverse populations. Similar eQTL analyses of LDL cholesterol and its subclasses have been conducted in non-African American groups, providing insights into the genetic determinants of lipid metabolism. For instance, studies by the Global Lipids Genetics Consortium explored genetic contributions across ancestries, including European and East Asian cohorts (Teslovich et al., 2010). Additionally, the Framingham Heart Study, predominantly involving European Americans (Zubair et al., 2016), offered valuable eQTL data for lipid traits. While not focused on African Americans, these studies provide a comparative context for assessing the uniqueness or commonality of our findings. Emphasizing the importance of including diverse populations in genetic research, they highlight the need to capture the full spectrum of genetic influences on complex traits like lipid metabolism.

Elevated LDL cholesterol is a key phenotype in the development of hypercholesterolemia, a major risk factor for cardiovascular diseases (CVD), which accounts for approximately 1 of every 5 deaths in the United States (U.S.) (Ference et al., 2017). It is estimated that abnormal LDL concentrations affect 70 million Americans and cost upwards of $35 billion dollars annually in health expenditures (Dieleman et al., 2020). While LDL’s role in hypercholesterolemia is well studied and widely used clinically, increasing evidence challenges the conventional view of LDL as the most relevant biomarker for hypercholesterolemia. Historically, the concentration of LDL has been estimated using their cholesterol content (LDL-C) (Dieleman et al., 2020). However, quantifying lipoproteins by their particle concentration rather than cholesterol concentration can improve risk assessment for CVD (Cantey and Wilkins, 2018; Liou and Kaptoge, 2020; Glavinovic et al., 2022). Specifically, large buoyant LDL and small dense LDL, are considered biomarkers of interest in CVD processes (Liou and Kaptoge, 2020). Buoyant LDL particles have a cholesterol rich core, and as such may be resistant to oxidation and possibly be anti-atherogenic (Liou and Kaptoge, 2020). Smaller and denser LDL particles are causal risk factors for CVD because of their greater susceptibility to oxidation and their permeability through the endothelium of arterial walls, which makes them pro-atherogenic (Ivanova et al., 2017), pro-thrombotic (Toth, 2014), and proinflammatory (Krychtiuk et al., 2015).

Similar to European Americans (EAs), approximately 1 in 4 African American (AA) adults (23%–29%) have elevated LDL concentrations (Tsao et al., 2022). Yet, AA are prescribed lipid-lowering medications less often (56.7%) compared with European Americans (EA; 67.7%) and are less likely to achieve LDL control (Dorsch et al., 2019). This study investigated a sample of African American from The GENomics, Environmental FactORs and the Social DEterminants of Cardiovascular Disease in African-Americans STudy (GENE-FORECAST).

## Material and methods

### Data description

GENE-FORECAST is a research platform that establishes a strategic, multi-omics systems biology approach amenable to the deep, multi-dimensional characterization of minority health and disease in AA. GENE-FORECAST is study designed to create a cohort based on a community sampling frame of self-identified, U.S.-born, AA men and women (ages 21–65) recruited from the metropolitan Washington D.C. area.

A description of the baseline characteristics of the GENE-FOREAST samples included in the analyses is outlined in Table 1. LDL cholesterol concentration was assessed as part of the fasting blood chemistry panel (overnight fast and no alcohol consumption for 24 h) from plasma collected in Lithium Heparin tubes. Among the 416 individuals examined, only 41 (10%) were receiving lipid-lowering medications, while 101 (24%) exhibited LDL levels equal to or exceeding 129 mg/dL. The *NMR LipoProfile* (Jeyarajah et al., 2006) lipoprotein particle test was employed to quantify both small and large LDL particles; each particle level is positively correlated with LDL cholesterol level. Age displayed a significant correlation with LDL levels, whereas factors such as gender, body mass index (BMI), and educational attainment did not. The study population consisted of a larger proportion of females than males, with over one-third of the individuals possessing a graduate-level education or higher. Hypertensives were defined as subject with systolic blood pressure (SBP) > 120 and/or diastolic blood pressure (DBP) > 80 and/or on high blood pressure medication or doctor diagnosed.

**TABLE 1:** Baseline characteristics of the 416 samples included in the differential expression analysis and their correlation with LDL cholesterol.

Characteristic	Mean or count	SD or proportion
LDL (mg/dL)	105	33
Small LDL (mg/dL)	477	333
Large LDL (mg/dL)	485	285
Lipid Lowering Medication		
No	376	90%
Yes	41	10%
BMI	32	7
Age (years)	48	12
Sex		
Female	289	69%
Male	127	31%
Education		
≤ high school	44	11%
Some vocational or college or technical school	125	30%
College graduate	112	27%
> Graduate	135	32%
Hypertension		
No	110	26%
Yes	306	73%
Systolic Blood Pressure	159	38
Diastolic Blood Pressure	76	10
HDL	59	16
Triglycerides	82	44
Type 2 Diabetes		
No	368	88.5%
Yes	47	11.5%

The transcriptome data consist of the messenger RNA sequencing (mRNA-seq) data of whole blood (buffy coat). Total RNA extraction was carried out using MagMAXTM for Stabilized Blood Tubes RNA Isolation Kit as recommended by vendor (Life Technologies, Carlsbad, CA). For library preparation, total RNA samples are concentration normalized, and ribosomal RNA (rRNA) is removed. Pooled libraries are bound to the surface of a flow cell and each bound template molecule is clonally amplified up to 1000-fold to create individual clusters. Illumina paired end 100 base pair sequencing was performed on HiSeq2000 analyzer (Illumina, USA) with an average sequencing depth of 50 million reads per sample. The mRNA expression was quantified using a bioinformatics pipeline developed by the Broad Institutes and used by the Genotype-Tissue Expression (GTEx). The pipeline is detailed in the GitHub software development platform (Broad Institute, 2015). The pre-analysis quality control (QC) procedures for mRNA sequencing data are detailed in Supplementary Material SM1, accompanied by graphical representations of each QC step. Briefly, the QC consisted of validation of target read depth, exclusion of lowly expressed transcripts, and subsequent normalization utilizing the Trimmed Mean of M-values (TMM) method (Robinson and Oshlack, 2010). Following these steps, principal component analysis was employed to detect any noteworthy outliers among samples and transcripts. After QC, the analysis incorporated 17,948 protein-coding mRNAs and 416 samples for whom LDL and LDL particle measurements were available.

The genotype data are from whole-genome sequencing (WGS) of a subset of 242 samples. DNA was extracted from whole blood Ethylenediaminetetraacetic acid (EDTA) tubes followed by picoGreen quantitation. Library preparation was done using *Whole Genome Small Insert PCR-Free*. The WGS samples were 151bp paired end sequenced on an Illumina NovaSeq6000 to a mean coverage of 30X. At the data preprocessing step, WGS reads were processed with the *Whole Genome Germline Variant Discovery* pipeline developed and used by the *Genomics Platform* at the Broad Institute. Reads were then aligned to the genome build Hg38 with Burrows-Wheeler Aligner and gVCF generation, joint genotype calling, and quality filtering were executed in accordance with GATK4 best-practices. A total of 8,581,606 SNPs with a minor allele frequency (MAF) ≥ 0.01 were considered for the analysis.

### Statistical analyses

**FIGURE 1:**
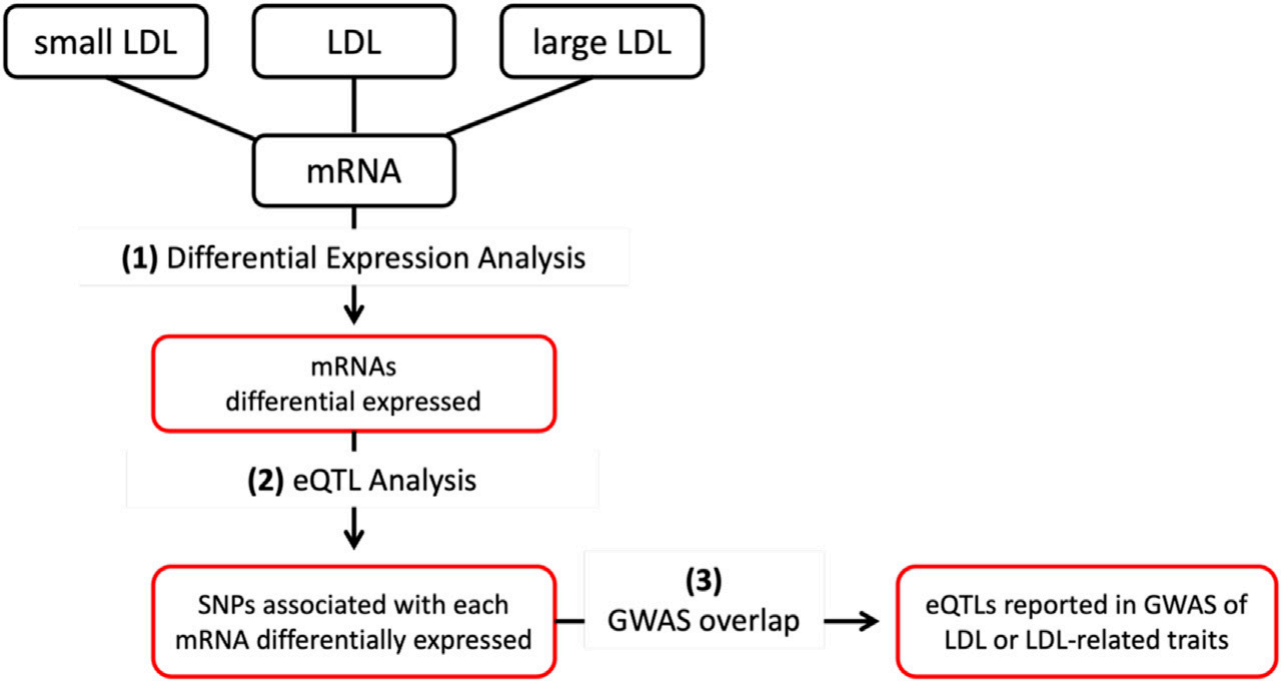
First, (1) mRNAs differentially expressed (DE) between the top and bottom tertiles of LDL, small LDL and large LDL are identified. Then, (2) eQTL analysis was conducted to identify cis-eQTLs associated with mRNAs DE by LDL, small and large LDL. Finally, cis-eQTLs reported in GWAS of LDL and LDL-related traits are identified.

The analytical steps of the project are depicted graphically in Figure 1 and detailed in the subsequent paragraphs. All the analyses were conducted on R version 4.3.1; R is a programming language and environment for statistical computing and graphics.

### Differential expression analyses

For each of the three differential expression (DE) analyses, the number of samples contrasted between the top and bottom tertiles are reported in the Supplementary Table SM1 along with the distribution of the covariates age, sex and education level. In summary, the DE analyses, involved a comparison of 137 samples in the lower tertile with 143 samples in the upper tertile for LDL, 139 samples versus 140 samples for small LDL, and 139 samples versus 140 samples for large LDL.

Differential expression (DE) analysis was conducted on a set of 17,948 protein-coding mRNAs, employing the R library *edgeR* (Robinson et al., 2010). This library fits a negative binomial model to the read counts of mRNAs and subsequently computes likelihood ratio tests for the coefficients within the model. More specifically, a gene-wise statistical test was conducted by fitting a negative binomial generalized log-linear model to the read counts (expression) of each gene. An empirical Bayes estimate of the negative binomial dispersion parameter was computed for each gene and that vector was used to set the dispersion parameter of the binomial model. The association was adjusted for age, sex and level of education (measure of socio-economic status). Statistical significance in differential expression between the upper and lower tertiles of LDL, small LDL, and large LDL was determined based on a false discovery rate-adjusted *p*-value ≤0.05. The DE analysis focused on the extremes of the lipid variables distribution, specifically the upper and lower tertiles, to enrich the subsequent eQTL analysis in novel variants with substantial effects.

### eQTL analysis

All SNPs with MAF ≥0.01 in the cis region (within 1Mb) of each mRNA differentially expressed by LDL, small LDL and large LDL were included in the eQTL analysis conducted using the R library *MatrixEQTL* (Shabalin, 2012). *MatrixEQTL* fits a regression model with mRNA expression as the outcome and additive genotypes as the independent variable. The regression was adjusted for age, sex and principal components (PCs) 1 to 6 to adjust for genetic ancestry admixture. A SNP is deemed a plausible eQTL if the *p*-value of the association with mRNA expression is statistically significant after adjusting for multiple testing (adjusted *p*-value ≤0.05).

### Overlap with GWAS variants associated with LDL and LDL-related traits

The eQTLs significantly associated with differentially expressed mRNAs were juxtaposed with genetic variants cataloged with genome-wide significance in the GWAS Catalogue database (version 1.0 as of 8th November 2023). The objective is to discern eQTLs identified in our analysis that have been previously reported in extensive investigations on LDL and LDL-related traits.

Finally, pathway and gene ontology enrichment analyses were conducted using a hypergeometric test. This involved sampling across the mRNA associated with eQTLs reported in GWAS, from the broader universe of Kyoto Encyclopedia of Genes and Genomes (KEGG) genes. The objective of these enrichment analyses was to discern pathways enriched in the list of mRNAs and establish connections to the GWAS traits.

## Results

### Differential expression analysis

**FIGURE 2:**
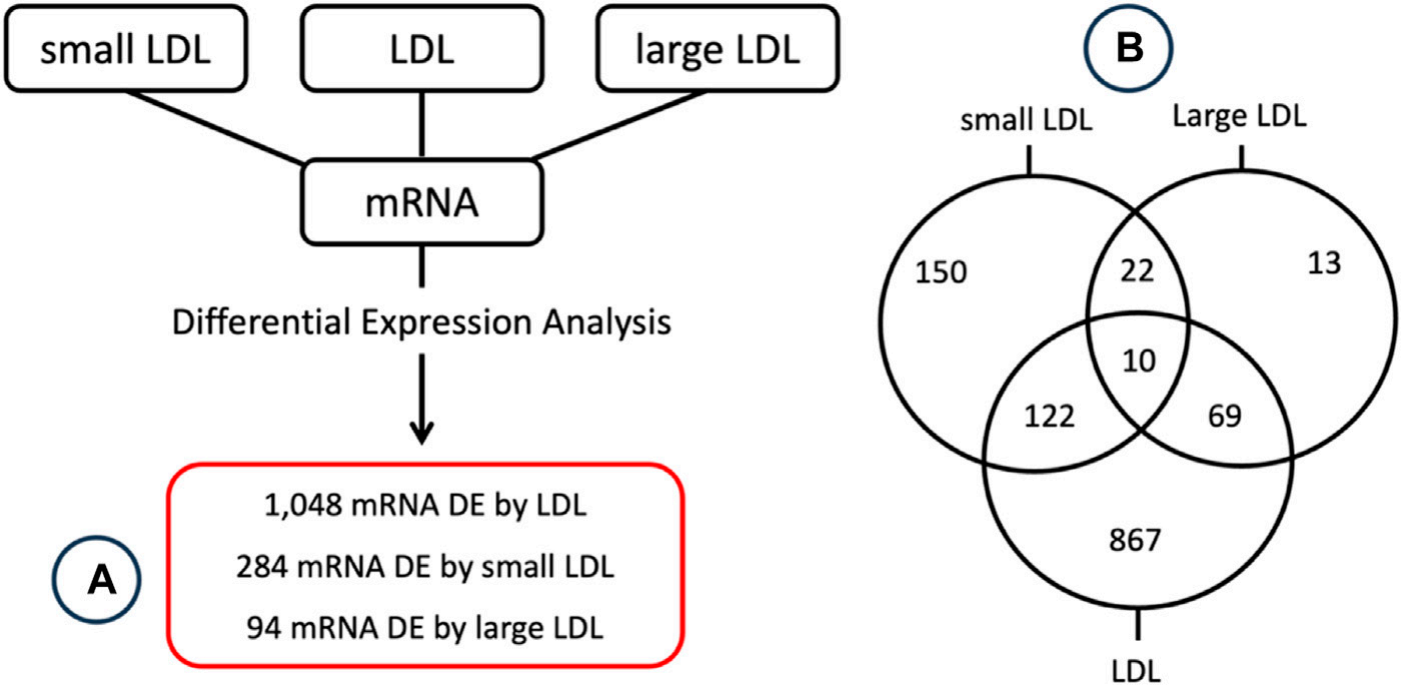
Graphical summary of the **(A)** differential expression analysis results along with the **(B)** number of differentially expressed genes overlapping between the 3 lists.

**FIGURE 3:**
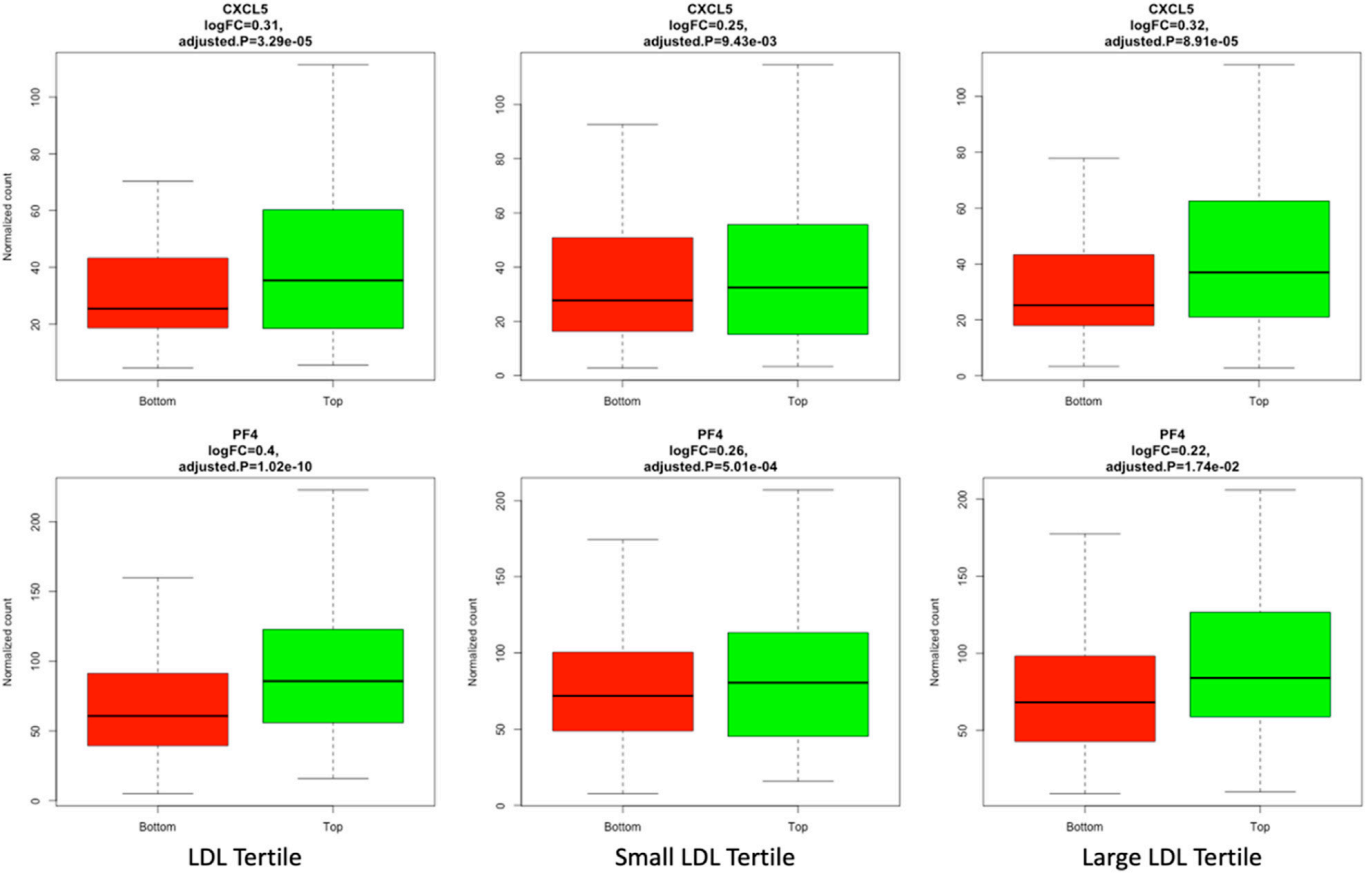
Plots of CXCL5 and PF4, 2 of the 10 mRNAs differentially expressed between top and bottom tertiles of LDL, small LDL and large LDL in respectively column 1, 2 and 3 of the graph.

Differential expression analysis was conducted to identify mRNAs that have a significant different level of expression between top and bottom tertiles of LDL, small LDL and large LDL. A total of 1048, 284 and 94 mRNA were significantly differentially expressed by LDL, small LDL and large LDL, respectively. The results are reported graphically in Figure 2, including the number of differentially expressed genes overlapping between LDL and small LDL (132), between LDL and large LDL (79), between small and large LDL (32) and those differentially expressed in all three analyses (10). The set of 10 mRNA that overlap between the 3 analyses includes OLFM4, CXCL5, PF4, CAMP, FLRT2, MUC12, DEFA1B, ITGB3, GOLGA6L22 and ENSG000002849. Two of those 10 associations are illustrated graphically in Figure 3 for LDL, small LDL and large LDL. The full list of significantly differentially expressed mRNAs is reported in Supplementary Table S1A.

### eQTL analysis and overlap with GWAS reported associations

**FIGURE 4:**
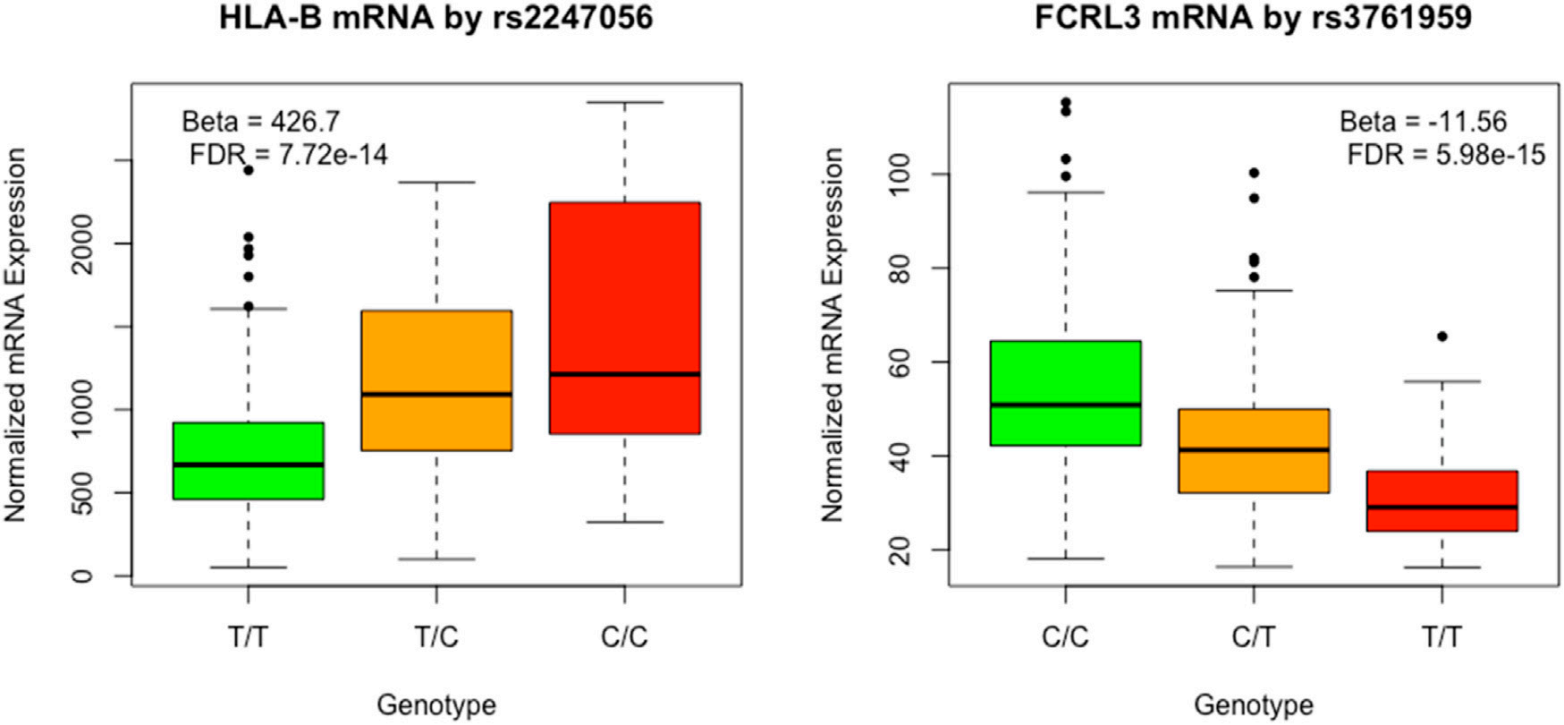
Plot of HLA-B and FCRL3 by respectively rs2247056 and rs3761959 associated with LDL cholesterol level in previous GWAS analyses.

The cis-eQTL analysis revealed a total of 9,950 associations between eQTL and mRNA transcripts, encompassing 6,955 distinct eQTL and 955 mRNA entities. A comprehensive summary of the eQTL reported in GWAS is provided in Supplementary Table S1B. Two of the eQTLs reported in GWAS of LDL are depicted graphically in Figure 4.

A subset of 101 eQTL identified in this analysis aligns with previously documented findings in GWAS studies of 40 LDL and LDL-related traits outlined in Table 2. A concise presentation of the count of eQTL affiliated with each mRNA and their respective associations with traits in GWAS is reported in Table 3.

**TABLE 2:** LDL and LDL-related traits associated with the 101 eQTL identified.

Trait	GWAS phenotypes
Low-Density Lipoprotein	Average diameter for LDL particles,
	Average diameter for VLDL particles,
	Free cholesterol levels in chylomicrons and extremely large VLDL, Free cholesterol to total lipids ratio in very small VLDL,
	LDL cholesterol, Total lipid levels in large LDL,
	Total lipid levels in LDL,
	Total lipid levels in small HDL,
	Total lipids in large LDL,
	Total lipids in LDL,
	Total lipids in small HDL
High-Density Lipoprotein (HDL)	HDL cholesterol,
	Free cholesterol levels in small HDL,
	Triglyceride levels in HDL,
	Triglyceride levels in medium HDL,
	Triglyceride levels in small HDL
Triglycerides	Triglycerides levels
Cholesterol	Cholesterol levels in large LDL,
	Total cholesterol levels,
	Total free cholesterol levels
Cardiovascular Disease (CVD)	Coronary artery disease,
	Coronary heart disease,
	Factor VIII levels
Anthropomorphic	Allometric body shape index,
	Adult body size,
	BMI,
	BMI (joint analysis main effects and physical activity interaction), BMI in physically active individuals,
	Fat-free mass,
	Hip circumference adjusted for BMI,
	Hip index,
	Waist circumference adjusted for BMI,
	Waist-hip index,
	Waist-hip ratio,
	Waist-to-hip ratio adjusted for BMI,
	Waist-to-hip ratio adjusted for BMI,
	Weight
Other	Circulating leptin levels in high cardiovascular risk

**TABLE 3:** Summary of the number of eQTL reported in GWAS of LDL and related traits (column 1), the mRNA the eQTLs are associated with in our eQTL analysis (column 2), and the GWAS traits reported as associated with the eQTLs (column 3–8).

eQTL count	mRNA	LDL	HDL	TG	Cholesterol	CVD	Anthropomorphic	Other
33	HLA-B	Average diameter for VLDL particles, Free cholesterol levels in chylomicrons and extremely large VLDL, Free cholesterol to total lipids ratio in very small VLDL, LDL cholesterol, Total lipid levels in large LDL, Total lipid levels in LDL		Triglycerides levels	Cholesterol levels in large LDL, Total cholesterol levels		A body shape index, Hip circumference adjusted for BMI, Hip index, Waist circumference adjusted for BMI, Waist-hip index, Waist-to-hip ratio adjusted for BMI, Weight	
30	HLA-DRB5	Average diameter for LDL particles				Coronary artery disease, Coronary heart disease, Factor VIII levels	A body shape index, Hip circumference adjusted for BMI, Waist circumference adjusted for BMI, Waist-hip index, Waist-hip ratio, Waist-to-hip ratio adjusted for BMI	Circulating leptin levels in high cardiovascular risk
25	HLA-DRB1	Average diameter for LDL particles, LDL cholesterol		Triglycerides levels		Coronary heart disease, Factor VIII levels	A body shape index, Hip circumference adjusted for BMI, Waist circumference adjusted for BMI, Waist-hip index, Waist-hip ratio, Waist-to-hip ratio adjusted for BMI	Circulating leptin levels in high cardiovascular risk
5	TAGLN	LDL cholesterol, Total lipid levels in large LDL, Total lipid levels in LDL, Total lipid levels in small HDL, Total lipids in large LDL, Total lipids in LDL, Total lipids in small HDL	Free cholesterol in small HDL, Free cholesterol levels in small HDL, HDL cholesterol	Triglycerides levels	Cholesterol in large LDL, Cholesterol levels in large LDL, Total cholesterol levels, Total free cholesterol levels			
4	BAG6						A body shape index, Hip circumference adjusted for BMI, Waist-hip index, Waist-hip ratio, Waist-to-hip ratio adjusted for BMI	
2	TTC38			Triglycerides levels				
2	SIDT2	LDL cholesterol	HDL cholesterol	Triglycerides levels	Total cholesterol levels			
2	CTSW		HDL cholesterol				Adult body size, BMI	
1	STAB1		HDL cholesterol	Triglycerides levels			A body shape index, Waist circumference adjusted for BMI, Waist-hip index, Waist-to-hip ratio adjusted for BMI	
1	CPSF1						Fat-free mass	
1	LYZ			Triglycerides levels				
1	NPRL3						BMI	
1	CD37	LDL cholesterol		Triglycerides levels	Total cholesterol levels			
1	CLIP2			Triglycerides levels				
1	LIMK1			Triglycerides levels				
1	GIT1					Coronary artery disease		
1	PADI2						Waist circumference adjusted for BMI	
1	MUC5B						BMI	
1	HMGA1						BMI, BMI in physically active individuals, Waist-to-hip ratio adjusted for BMI, Waist-to-hip ratio adjusted for BMI	
1	MAN2C1						Weight	
1	SP2						BMI	
1	GATAD2A			Triglycerides levels				
1	CD151						BMI	
1	POM121			Triglycerides levels				
1	MUC2						BMI	
1	AIF1						A body shape index, Hip circumference adjusted for BMI, Waist-hip index, Waist-to-hip ratio adjusted for BMI, Weight	
1	ACKR1			Triglycerides levels				
1	MUC5AC						BMI	
1	CKLF						BMI	
1	HLA-DPB1						Hip circumference adjusted for BMI	
1	CTXN2						BMI	
1	CD300H		Triglyceride levels in HDL, Triglyceride levels in medium HDL, Triglyceride levels in small HDL					

The aforementioned 101 eQTL are involved in 127 significant eQTL-mRNA associations, implicating 32 distinct mRNA transcripts. Within these associations, the predominant location of the eQTL is upstream of the mRNA (169 instances), followed by downstream positioning (113 instances); in four cases, the eQTL is an exonic non-synonymous SNP. A total 92 of the 101 eQTL are common (MAF ≥0.05); for the remaining 9 eQTL, the MAF ranges from 0.013 to 0.042. Importantly, all 101 eQTLs are characterized as common in the context of the GWAS reports. Furthermore, only two of the 101 eQTL, namely rs3094219 and rs3094214, situated on chromosome 6, are in linkage disequilibrium.

Pathway and gene ontology (GO) enrichment analysis, across the 32 mRNA associated with eQTL reported in GWAS, revealed a number of pathways and GO terms relevant for the GWAS traits; the results are summarized in Tables 4, 5.

**TABLE 4:** Results of pathway enrichment analysis across the 32 mRNA associated with eQTL reported in GWAS of LDL and related traits.

Pathway ID	Subcategory	Pathway description	*p*-value	mRNAs in pathway
hsa04940	Endocrine and metabolic disease	Type I diabetes mellitus	1.64e-05	HLA-DRB1, HLA-DRB5, HLA-B, HLA-DPB1
hsa05320	Immune disease	Autoimmune thyroid disease	2.88e-05	HLA-DRB1, HLA-DRB5, HLA-B, HLA-DPB1
hsa05416	Cardiovascular disease	Viral myocarditis	5.92e-05	HLA-DRB1, HLA-DRB5, HLA-B, HLA-DPB1
hsa04612	Immune system	Antigen processing and presentation	9.03e-05	HLA-DRB1, HLA-DRB5, HLA-B, HLA-DPB1
hsa04672	Immune system	Intestinal immune network for IgA production	4.37e-04	HLA-DRB1, HLA-DRB5, HLA-DPB1
hsa04145	Transport and catabolism	Phagosome	7.33e-04	HLA-DRB1, HLA-DRB5, HLA-B, HLA-DPB1
hsa04514	Signaling molecules and interaction	Cell adhesion molecules	7.60e-04	HLA-DRB1, HLA-DRB5, HLA-B, HLA-DPB1
hsa05321	Immune disease	Inflammatory bowel disease	7.60e-04	HLA-DRB1, HLA-DRB5, HLA-DPB1
hsa04658	Immune system	Th1 and Th2 cell differentiation	1.53e-03	HLA-DRB1, HLA-DRB5, HLA-DPB1
hsa04970	Digestive system	Salivary secretion	1.53e-03	MUC5B, MUC5AC, LYZ
hsa04659	Immune system	Th17 cell differentiation	2.01e-03	HLA-DRB1, HLA-DRB5, HLA-DPB1
hsa05322	Immune disease	Systemic lupus erythematosus	3.60e-03	HLA-DRB1, HLA-DRB5, HLA-DPB1
hsa04657	Immune system	IL-17 signaling pathway	1.79e-02	MUC5B, MUC5AC
hsa00511	Glycan biosynthesis and metabolism	Other glycan degradation	3.78e-02	MAN2C1
hsa04940	Endocrine and metabolic disease	Type I diabetes mellitus	1.64e-05	HLA-DRB1, HLA-DRB5, HLA-B, HLA-DPB1

**TABLE 5:** Results of GO enrichment analysis across the 32 mRNA associated with eQTL reported in GWAS of LDL and related traits.

GO ID	Description	Adjusted *p*-value	mRNA
GO:0042605	peptide antigen binding	6.03e-05	HLA-DRB1, HLA-DRB5, HLA-B, HLA-DPB1
GO:0023026	MHC class II protein complex binding	3.46e-04	HLA-DRB1, HLA-DRB5, HLA-DPB1
GO:0023023	MHC protein complex binding	6.07e-04	HLA-DRB1, HLA-DRB5, HLA-DPB1
GO:0042277	peptide binding	2.36e-03	HLA-DRB1, HLA-DRB5, HLA-B, HLA-DPB1, POM121
GO:0003823	antigen binding	2.36e-03	HLA-DRB1, HLA-DRB5, HLA-B, HLA-DPB1
GO:0033218	amide binding	4.69e-03	HLA-DRB1, HLA-DRB5, HLA-B, HLA-DPB1, POM121
GO:0004553	hydrolase activity, hydrolyzing O-glycosyl compounds	7.00e-02	LYZ, MAN2C1

## Discussions

Systems genetics integrates genetic information with molecular endophenotypes, such as the transcriptome, to overcome the challenge of understanding the mechanisms behind the association between genetic variants and diseases (Civelek and Lusis, 2014). This is achieved through a process of elucidating the interconnections and discerning how a genetic variant exerts its influence on a given phenotype (Wierbowski et al., 2018). Particularly pivotal in this context are expression quantitative trait loci (eQTLs) that co-locate with loci identified through genome-wide association studies (GWAS), as they play a crucial role in bridging the gap between genetic variants and the pertinent gene expression alterations associated with the GWAS-trait (Liu et al., 2022). Our study, conducted within a cohort of African American individuals, elucidates an intricate genetic landscape that influences LDL cholesterol and its subclasses. These findings significantly contribute to the burgeoning understanding of the genetic determinants underpinning lipid metabolism. In our exploration of the genetics of lipid metabolism, our discourse has centered on genes exhibiting noteworthy expression alterations in whole blood, with a particular emphasis on those biologically pertinent to the targeted phenotypes. The selection of whole blood as the analytical tissue is deliberate, owing to its ready accessibility and its role as a reflective medium of the body’s physiological status. It affords a comprehensive perspective on systemic gene expression modifications linked to lipid metabolism.

We identified distinct molecular profiles associated with LDL and LDL particles: 1,048 differentially expressed mRNAs for LDL, 284 for small LDL, and 94 for large LDL. Overlapping patterns include 132 mRNAs common to LDL and small LDL, 79 to LDL and large LDL, and 32 between small and large LDL, implying shared pathways in their roles in lipid metabolism and cardiovascular risk. Notably, 10 mRNAs were consistently differentially expressed across all three analyses, suggesting potential key regulatory nodes in lipid-related pathways. Cis-eQTL analysis unveiled 9,950 associations, indicating a significant genetic influence on mRNA expression levels. Integration with GWAS data enhanced the credibility of these eQTLs, with 101 aligning with previously reported associations.

### Associations of MHC-Related genes with LDL cholesterol, cardiovascular disease, and immune responses

The study revealed significant associations between specific mRNAs (e.g., HLA-DRB1, HLA-DRB5, HLA-B, HLA-DPB1) highlighted in both our pathway and Gene Ontology (GO) enrichment analyses. The immune system has been increasingly recognized for its role in metabolic processes (Zmora et al., 2017), including lipid metabolism. The MHC-related genes discussed are expressed in blood and have known associations with lipid levels and cardiovascular risk, making them relevant for whole blood analysis (Zhang et al., 2023). These genes are statistically significant in our eQTL analysis and showed biological plausibility in their roles related to lipid metabolism, cardiovascular risk, or immune response, which are all processes reflected in whole blood dynamics. Identified through eQTL analysis, these mRNAs are linked to traits reported in GWAS, such as LDL cholesterol levels and cardiovascular disease markers. Recent insights into the regulatory role of miRNAs in lipid metabolism, particularly miR-122 and miR-33, add an additional layer to our understanding of post-transcriptional regulation in lipid homeostasis. As highlighted in recent literature, miR-33’s multifunctional roles, which extend to macrophage activation and vascular homeostasis, complement our findings by providing potential mechanistic links between lipid metabolism and cardiovascular health (Aryal et al., 2017).

Notably, genes like HLA-DRB1 and HLA-DRB5, situated in or near the human major histocompatibility complex (MHC) on chromosome 6 (Caillier et al., 2008), exhibit associations with immune diseases (Jacobson et al., 2008) (e.g., autoimmune thyroid disease) and cardiovascular traits (Klein and Danzi, 2016) (e.g., coronary artery disease). The MHC region has been associated with lipid traits in multiple population studies, suggesting that genetic variants in this region may influence lipid levels systemically (Kathiresan et al., 2008). Furthermore, the involvement of lncRNAs in lipid metabolism, as evidenced by their presence on HDL in individuals with familial hypercholesterolemia, aligns with our observations of significant genetic associations in lipid-related pathways. Particularly, the association of HDL-lncRNA LEXIS with lipoprotien levels and vascular impairment underscores the importance of exploring non-coding RNAs in the context of cardiovascular risk stratification (Scicali et al., 2024).

Within the HLA-B region, 33 eQTLs were associated with reported GWAS traits including LDL, HDL, TG, total cholesterol levels, as well as cardiovascular disease (CVD), and anthropomorphic measures. This aligns with existing knowledge about antigen presentation, emphasizing the critical roles of MHC-related genes in immune response and their implications in inflammatory, autoimmune diseases (Fernando et al., 2008), and cardiovascular risk (Porto et al., 2005). Recent genome-wide association studies have linked genetic variants in the MHC region to cardiovascular risk (Dehghan, 2018).

A particularly intriguing finding is the upregulation of the HLA-DRB1 gene associated with elevated LDL cholesterol levels. The observed upregulation suggests a potential compensatory or reactive mechanism in the body in response to increased LDL levels (Wysocki et al., 2020). Research on the HLA-DRB1 gene, particularly HLA-DRB1*04:01 (a particular variant of HLA-DRB1), shows its impact on LDL and HDL cholesterol levels. Blackler et al. reported that the DR4tgLdlr^−/−^ mice, mice generated by crossing HLA-DRB1*04:01 transgenic mice with Ldlr−/− (LDL knockout mice), showed comparable atherosclerosis levels to Ldlr−/− mice on a high-fat diet, despite their lower LDL levels (Blackler et al., 2023). Their research suggests HLA-DRB1*04:01 might increase oxidized LDL (OxLDL), a more damaging LDL variant, potentially due to systemic inflammation mechanisms, consequently heightening the risk of cardiovascular complications (Blackler et al., 2023).

### SIDT2 gene implications in LDL cholesterol and lipid metabolism

The SIDT2 (SID1 transmembrane family, member 2) gene, known for its involvement in cellular double-stranded RNA (dsRNA) uptake and studied in viral RNA transport and immune responses, exhibits a significant upregulation in individuals with higher LDL cholesterol (Qian et al., 2023). The SIDT2 gene has been implicated in lipid metabolism through its role in autophagy and transport of cholesterol and triglycerides. Additionally, our eQTL analysis links SIDT2 to lipid metabolism traits through its association with a SNP that decreases its expression. This SNP (rs236911) is implicated in GWAS studies with triglycerides and total cholesterol levels, emphasizing its regulatory role in lipid metabolism (Hoffmann et al., 2018). The SNP is a non-coding exonic variant located in PCSK7, a gene whose perturbations have been linked to dyslipidemia (Dongiovanni et al., 2019). The combined evidence from eQTL and GWAS underscores the significance of the SNP in lipid-related traits, providing insights into the interplay of genetic variations, gene expression, and lipid metabolism.

These findings align with previous research highlighting SIDT2’s crucial role in lipid autophagy and metabolism, especially in cholesterol and triglyceride transport in mammalian cells, notably the liver (Leon-Mimila et al., 2021). SIDT2-knockout experiments further support its influence on lipid traits. SNPs in SIDT2 have significant associations with LDL levels in GWAS and gene expression studies (Chen et al., 2018). The observation that SIDT2 is primarily associated with triglycerides in human cohorts is consistent with SIDT2 knockout mice, indicating its pivotal role in lipid metabolism and potential impact on cardiovascular risk factors (Leon-Mimila et al., 2021).

The literature on the role of SIDT2 in liver cells supports its systemic effect on lipid levels, as the liver is a central organ in lipid metabolism (Chen et al., 2018). Our findings in whole blood are consistent with these roles, suggesting that alterations in SIDT2 expression could reflect systemic changes in lipid handling. This integration of human cohort data with experimental models underscores SIDT2’s importance as a research target for understanding and potentially managing lipid-related disorders (Song et al., 2023).

### Differential expression of TTC38 in relation to LDL cholesterol levels and population-specific genetic variation

Beyond the notable findings pertaining to SIDT2, our investigation has identified the gene TTC38 (tetratricopeptide repeat domain 38) as differentially expressed in relation to LDL cholesterol levels. The data indicate a significant upregulation of TTC38 in individuals with elevated LDL cholesterol. The observed upregulation of TTC38 in the context of heightened LDL cholesterol levels suggests a potential regulatory role in lipid profiles, contributing to the comprehension of genetic influences on lipid metabolism and associated cardiovascular risks. In contrast to the extensively studied SIDT2, which is recognized for its role in lipid autophagy and metabolism, the specific functions, and mechanisms of TTC38 in lipid homeostasis remain less elucidated.

The association of TTC38 with lipid metabolism is further underscored by our findings and supported by existing literature, indicating a potential role in regulating lipid profiles and cardiovascular health. Emphasizing the gene’s significance, a African ancestry-specific eQTL, rs6008712, showed association with TTC38 expression in African Americans (AA) and displayed a suggestive trend in the broader GTEx liver cohort. This population-specific SNP (present only in African and admixed-American, in the 100 Genomes project) highlights ethnic-specific genetic regulation of TTC38 (Zhong et al., 2020). The observation that TTC38’s expression is influenced by genetic variants, particularly in AAs, underscores the complex interplay between genetic background and lipid metabolism. This emphasizes the necessity for more inclusive genetic studies for advances in precision medicine and understanding ethnic disparities in disease prevalence and drug response.

While the precise functional role of TTC38 in lipid metabolism remains incompletely elucidated, its significant association with LDL cholesterol in our study, coupled with the identification of specific eQTLs in diverse populations, positions TTC38 as a promising candidate gene for further exploration in the context of lipid-related disorders and cardiovascular risk management.

While the specific functions of TTC38 in lipid metabolism are not fully understood, its association with LDL cholesterol in our study points to a potential regulatory role. Given that blood lipids are indicators of metabolic health, genes like TTC38 that show differential expression in relation to lipid levels are of systemic interest. Recent research demonstrates that genetic variants associated with blood lipid levels can affect cardiovascular risk, further supporting the relevance of our findings in whole blood (Smith et al., 2014).

Finally, a broader group of genes, including CTSW, STAB1, CD37, CLIP2, LIMK1, GATAD2A, POM121, ACKR1, CPSF1, NPRL3, MUC5B, HMGA1, MAN2C1, SP2, CD151, MUC2, AIF1, MUC5AC, CKLF, HLA-DPB1, CTXN2, and CD300H, unveils a nuanced interplay through their associations with lipid metabolism and anthropomorphic traits. CTSW, notably linked to HDL cholesterol and diverse body size metrics, hints at a potential involvement in lipid processing and anthropometric characteristics (Joehanes et al., 2013). The broader group of genes discussed, including CTSW and STAB1, have been linked to lipid processing and cardiovascular health. For example, CTSW has been studied for its role in HDL cholesterol metabolism and its potential impact on atherosclerosis (Cheng et al., 2023), while STAB1 has been implicated in the clearance of atherogenic lipoproteins (Verwilligen et al., 2022). These associations reinforce the systemic nature of blood as a reflection of lipid metabolic health. Additionally, the eQTL rs11205277 on chromosome 1 demonstrates a significant impact on LDL cholesterol levels through its association with the ADAMTSL4 gene. This variant’s influence extends to body fat distribution, specifically waist circumference adjusted for BMI, evident in both the general population and non-smokers. Its consistent correlation with these traits across various GWAS studies (Gudbjartsson et al., 2008; Justice et al., 2017; Galvan-Femenia et al., 2018), suggests a potential role in lipid metabolism and anthropometric variations. Our findings illuminate a complex genetic architecture underlying lipid metabolism and body shape index. The eQTL’s significant association with LDL cholesterol aligns with patterns observed in other genes, highlighting genetic influences on lipid levels and cardiovascular risk factors (Willer and Mohlke, 2012). The consistent correlation with waist circumference and BMI, as discussed earlier regarding the genetic basis of anthropometric traits, reinforces the multifaceted nature of genetic contributions to lipid metabolism and body shape (Shen et al., 2006). This underscores the significance of considering a broad spectrum of genetic variants to comprehend these intricate traits comprehensively.

## Conclusion

In summary, our study employs a systems genetics approach, integrating genetic information with transcriptomic insights from eQTL analysis, to unravel the complex relationships between genetic variants and phenotypes and provide a detailed genetic landscape influencing lipid metabolism (Allayee et al., 2023). Our investigation delineates an approach for unraveling variant-trait relationships within GWAS by (a) establishing the relationships that exist between gene expression patterns and traits, (b) identifying genetic variants linked to the genes associated with these traits, and (c) substantiating the correlation between the genetic variants and the traits in the context of GWAS studies. While the present analysis is specifically centered on LDL cholesterol and its subclasses, the outlined approach holds applicability across a broad spectrum of traits.

While our study offers valuable insights, it is crucial to acknowledge its limitations. The intricate nature of lipid metabolism implies the involvement of numerous genetic factors beyond the scope of this study. Subsequent research endeavors should seek to validate these SNP associations using both *in vitro* and *in vivo* models to comprehensively understand their roles in gene-trait relationships. The differential analysis was conducted with mRNA expression from whole blood which is composed of multiple cell types. Hence the differential expression differential could be biased by potential differences in cell type composition.

## Data Availability

The datasets presented in this article cannot be publicly shared due to privacy restrictions. Requests to access the datasets should be directed to the corresponding author.
