# Supplementary material for: Leveraging the transcriptome to further our understanding of GWAS findings: eQTLs associated with genes related to LDL and LDL subclasses, in a cohort of African Americans
Source: Front Genet. 2024 Feb 7;15:1345541. doi: 10.3389/fgene.2024.1345541 (PMC10879560; doi:10.3389/fgene.2024.1345541)
Supplement: Supplementary file 1 [file DataSheet1.pdf]

Summary of QC for the mRNA-Seq data

A set of 498 samples from the GENE-FORECAST cohort underwent sequencing at the National Institutes of Health Sequencing Center (NISC). Among these, a subset of 416 samples had their *NMR LipoProfile* lipoprotein particles measured, forming the basis for our analytical investigation. It is noteworthy that the quality control (QC) procedures detailed herein were executed across the entirety of the 498 samples and all the mRNA transcript biotypes captured in the sequencing assay. Figure 1 below provides a succinct depiction of the QC process, while subsequent paragraphs described the individual steps involved.

Figure 1: Overview of the QC steps undertaken.

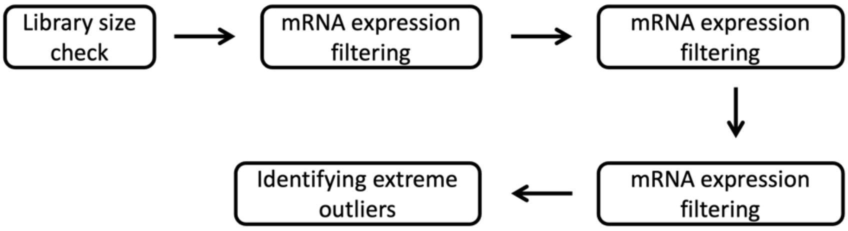

The target sequencing depth requested from NISC was 50 million reads. Figure 2 shows that an average of library size of 50,730,015 reads was achieved per samples. This resolution allows for exploratory works like the one presented in this project.

Figure 2: Library size across the entire set of samples (each bar is a sample) and mRNA transcripts

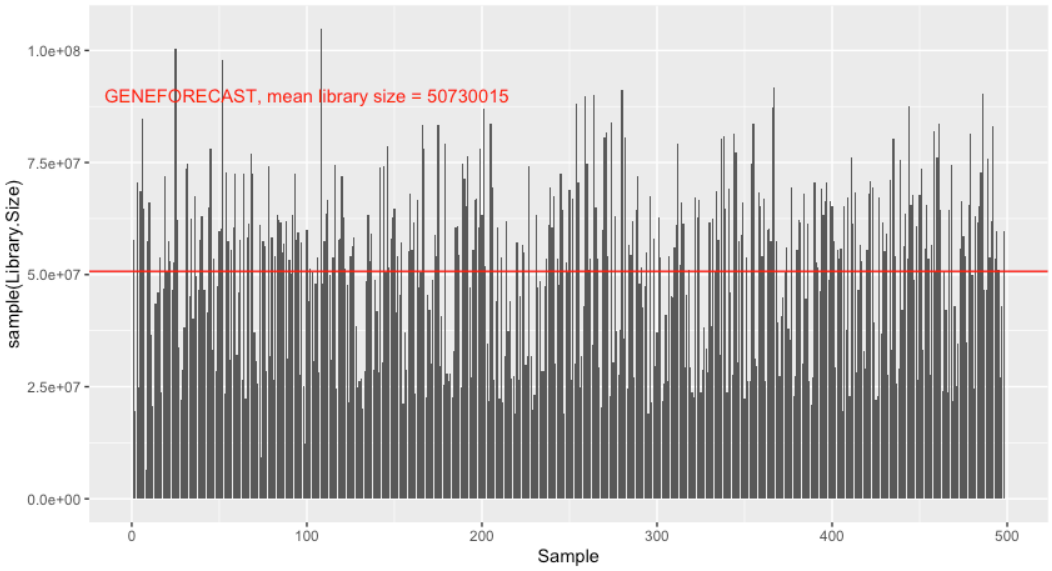

Next, mRNA transcripts not expressed in any of the samples, i.e. transcripts with read counts = 0 in all the samples, were removed. A set of 5,562 transcripts were excluded from a total of 60572 transcripts which include protein coding mRNAs as well as lncRNAs, pseudogenes, scRNAs, and other biotypes.

Subsequently, transcripts with a low expression across the entire dataset were also excluded. Lowly expressed genes may have higher variability in their measurements, leading to increased noise in the data. Therefore, by removing them we increase the statistical robustness of the analysis. However, there

is no set guidelines to determine the threshold but rather the threshold is based on the characteristics of the dataset and the goals of the analysis. For our analysis, after evaluating different cut-offs and given the large sample size (more than half the sample size of the GTEx study which until very recently was the reference) the optimal threshold was set to 0.0394 count-per-million (CPM) which is approximately equivalent to 2 read counts in a sample of average library size. A total of 18,691 mRNA transcripts were excluded for not achieving that threshold in more than 90% of the samples. The remaining 36,319 transcripts were carried forward. Figure 3 shows the read count distribution per sample before (Figure 3A) and after (Figure 3B) filtering out non-expressed and lowly expressed transcripts.

Figure 3: Distribution of expression (logCPM) before (A) and after (B) filtering lowly expressed transcripts.

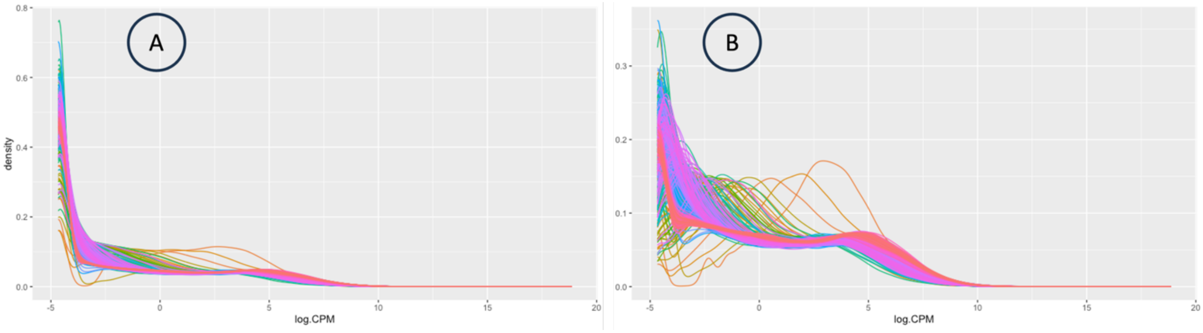

The data were then normalized using the Trimmed Mean of M-values (TMM) method. TMM normalization is commonly used and proves highly beneficial in the analysis of RNA-seq data by mitigating challenges associated with variations in library sizes and minimizing the influence of both highly variable and lowly expressed genes. This method offers a sturdy and efficient means of comparing gene expression levels across samples, facilitating enhanced precision in the detection of differentially expressed genes.

Finally, principal component analysis (PCA) was undertaken with the filtered and normalized data to identify and exclude extreme outliers among the 36,319 transcripts and 498 samples. In both the sample and transcript PCA, PC1 explained most the variance in the data (> 60 % variance explained), and therefore only samples or mRNA that are extreme outliers in the PC1 dimension were excluded. Figure 4 shows a plot of the two first PCs, the results indicate 3 transcripts as outliers and those were excluded.

Figure 4: PCA plots for the samples (left) and transcripts (right) with the names of the 3 mRNAs excluded.

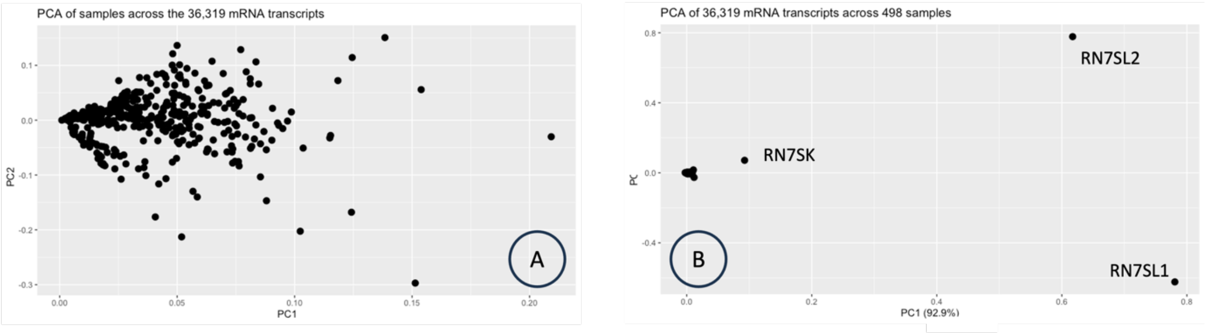

Number of samples in the top and bottom tertiles compared

Table 1: Number of observations in the top and bottom tertile groups contrasted in the differential expression analysis and distribution of covariates.

LDL Type	Variable	Bottom Tertile		Top Tertile		P-Value
		Mean or Count	SD or Proportion	Mean or Count	SD or Proportion	
LDL	N	137		143		
	Age (years)	47	13	51	10	0.003
	Sex (0 / 1)	95 / 42	69.3% / 30.7%	98 / 45	68.5% / 31.5%	0.99
	Education level (0 / 1 / 2 / 3)	14 / 41 / 35 / 47	10.2% / 29.9% / 25.5% / 34.3%	17 / 43 / 37 / 46	11.9% / 30.1% / 25.9% / 32.2%	0.96
Small LDL	N	139		140		
	Age (years)	47	13	49	10	0.14
	Sex (0 / 1)	106 / 33	76.3% / 23.7%	82 / 58	58.6% / 41.4%	0.003
	Education level (0 / 1 / 2 / 3)	7 / 39 / 38 / 55	5% / 28.1% / 27.3% / 39.6%	19 / 52 / 36 / 33	13.6% / 37.1% / 25.7% / 23.6%	0.005
Large LDL	N	139		140		
	Age (years)	47	12	49	11	0.12
	Sex (0 / 1)	87 / 52	62.6% / 37.4%	110 / 30	78.6% / 21.4%	0.005
	Education level (0 / 1 / 2 / 3)	14 / 45 / 37 / 43	10.1% / 32.4% / 26.6% / 30.9%	12 / 39 / 40 / 49	8.6% / 27.9% / 28.6% / 35%	0.78

Sex was coded as follow:
0 = Female
1 = Male
Education level was coded as followed:
0 = '≤ high school'
1 = 'Some vocational or college or technical school'
2 = 'College Graduate'
3 = 'Graduate'
